# Enhancing core components for a digital “healthy eating” resource in early childhood and care settings: staff’s perceptions and needs

**DOI:** 10.1186/s12889-024-20456-2

**Published:** 2024-10-30

**Authors:** Sissel H. Helland, Tormod Bjørkkjaer, Kristine Vejrup, Nina C. Øverby

**Affiliations:** 1 Department of Nutrition, and Public Health, Faculty of Health and Sport Sciences, University of Agder, Kristiansand, Norway; 2 Norwegian Armed Forces Joint Medical Services, Institute of Military Epidemiology, Oslo, Norway

**Keywords:** Digital, Early education and care, Healthy eating, Program, Nutrition

## Abstract

**Background:**

In response to the growing need for effective programmes to promote healthy eating habits among children in real-world settings, we conducted a qualitative study. Our aim was to explore the content-related needs of early childhood education and care (ECEC) staff regarding the core components of an upcoming digital “healthy eating” resource. This resource, designed for real-world implementation through the *Nutrition Now* project, seeks to enhance children’s nutrition and health within ECEC settings.

**Methods:**

Twelve individual semi-structured interviews with ECEC staff in a Norwegian municipality were conducted. Subsequently, we conducted two focus group discussions, involving five participants, to encourage reflection on the preliminary findings from the individual interviews. Qualitative thematic analysis was conducted using Nvivo software. Data from the interviews and focus groups were transcribed verbatim and analysed thematically to identify and summarise staff’s subjective experiences and reasoning.

**Results:**

Six themes were identified for the development of the upcoming digital “healthy eating” resource: (1) *A comprehensive recipe bank*,* and menu suggestions*, (2) *Tips for easy and quick cooking*,* simple hygiene and allergy management*, (3) *Age-appropriate food learning ideas*, (4) *Strategies for mealtime learning and engagement*, (5) *Alignment with the national ECEC curriculum*, and (6) *Parent-friendly access and content.*

**Conclusions:**

This research provides valuable guidance and informs the adaptation of an expert-led digital “healthy eating” intervention to better suit ECEC staff and the ECEC context, consequently enhancing its feasibility and effectiveness.

**Trial registration:**

This study was not registered in a trial registry as it is not a clinical trial or intervention study but serves as a pilot for the Nutrition Now study, trial identifier ISRCTN10694967 (https//doi.org/10.1186/ISRCTN10694967), registration date 19/06/2022.

## Background

A healthy diet is essential for children’s development and lifelong health and well-being. In Western societies, many children attend early childhood education and care (ECEC) from early childhood until they start school, providing a unique opportunity to cultivate healthy food habits [1], encourage acceptance of a variety of healthy foods [2–4], and promote social interaction during mealtimes [5]. Previous studies have shown that implementing “healthy eating” programmes in ECEC settings, called ‘daycare’, ‘nursery school’ or ‘kindergartens’ in some countries, can have positive impacts on the diets of preschoolers [6]. Furthermore, multi-component programmes targeting the environment, staff, children, and parents have been shown to be effective in promoting healthy eating habits among children enrolled in ECEC, as demonstrated through randomised controlled trials (RCTs) [1, 6].

Importantly, researchers, practitioners, and policymakers face a universal challenge in translating the content of such effective “healthy eating” programmes into daily educational and health-promoting practices within ECEC settings [6]. This evidence-to-practice gap, where efficacious programmes fail to be implemented and scaled-up in real-world settings, represents a significant waste of resources [7] and has paved the way for implementation science. Implementation science refers to the use of strategies to adopt and integrate programmes into practice, resulting in changes in practice patterns within a given setting [8].

Effective programmes should focus on core components, which are the essential elements that staff should engage with during implementation [9]. This is necessary to ensure sustained delivery while allowing for adaptation and maintaining fidelity to the programme [9–11]. These core components often include aspects such as (1) ECEC’s menus and cooking practices, (2) policies and guidelines, (3) age-appropriate health-promoting curricula, and (4) parent cooperation. Additionally, staff training should be an integral part of working with the content of these core components [6]. To understand how a programme might function in a real-world setting, it is essential to include users. This means understanding the local context, identifying additional needs, and making any necessary adjustments based on these factors [6]. User involvement and qualitative formative research are essential to understanding user perspectives, attitudes, and experiences, allowing a better grasp of the complex aspects of human behaviour that affect programme success [12]. Failing to adapt programmes and implementation to the context when scaling up can result in a reduction in effectiveness, known as “the voltage drop.” This drop in effectiveness occurs when the programme is not adapted to local needs and conditions [13].

In this context, ECEC centres in Norway provide childcare services for children aged 0–5 years. In Norway, children may either bring packed lunches from home or be provided with meals prepared at the ECEC, with some institutions adopting a combination of both approaches [14]. The quality of meals in these centres depends on several factors, including the available budget, as highlighted in a cross-sectional audit by Sambell (2020), which examined food expenditure and its impact on compliance with dietary guidelines [15]. Other factors include adequate kitchen facilities [16, 17], the adequacy of staffing [18, 19], as well as the food and nutrition knowledge of the staff and their ability to plan healthy menus for children. ECEC teachers in Norway lead the development and implementation of educational plans in line with national curriculum guidelines and mentor other staff in pedagogical work [20]. In centres without a dedicated cook, teachers and staff typically take turns preparing meals [16]. Unfortunately, current ECEC teacher education programmes in Norway do not emphasise the promotion of healthy eating habits (Universities Norway, 2018). In Norway, relatively few ECEC centres employ professional chefs [16]. Although these chefs may be skilled in preparing large quantities of food, they may not possess the necessary expertise to provide nutritionally balanced meals.

Previously, we conducted a multi-component programme in Norwegian ECEC, targeting both environmental and individual-level determinants of healthy eating behaviours. Our digital “healthy eating” programme, *Pre-schoolers’ Food Courage 2.0* [21], aimed at children aged 1 year, promoted the consumption of specific vegetables among the children participating in the programme [22]. The staff were asked to carry out four components: (1) prepare and serve hot lunch dishes for the children three times a week, (2) introduce selected vegetables during playful sensory sessions with the children, (3) follow health-promoting advice during mealtimes, and (4) communicate with parents [23]. The subsequent process evaluation revealed that ECEC teachers found the programme educational and inspiring [19]. Some of the centres that did not have a chef found it challenging to find time to prepare hot lunch dishes for the children three times a week. This prompted changes in staff pedagogical practices and food culture within ECEC settings. Specifically, the ECEC teachers noted that their awareness of the importance of exposing children aged one year to novel foods increased. They aimed to include new recipes, allocate more time for cooking than they had previously, and continue with playful sensory sessions with the children [19]. Based on these results, we adjusted certain core components to implement them on a larger scale to benefit various user groups and society.

Building on the success of *Pre-schoolers’ Food Courage 2.0*, we aim to incorporate it into a new digital resource called the *Nutrition Now* resource, which is designed to promote a healthy diet early in life across various settings. This resource is to be implemented in several municipalities in Norway [24]. To ensure high fidelity in implementation, our qualitative study explores the perceptions and needs of staff in a specific municipality regarding the content of the forthcoming digital “healthy eating” resource. This study centres around the following key research question: *What are the perceptions and needs of staff working in ECEC regarding core components of the upcoming digital “healthy eating” resource?*

## Methods

### Design

The current study is part of the *Nutrition Now project*, which involves implementing a digital programme in both ECECs and health care centres within a municipality in southern Norway [24]. This pre-study concerns the ECEC setting, aiming to enhance the content of the core components of the digital resource before full implementation. As an initial step in *Nutrition Now*, during spring and autumn 2020, we conducted qualitative interviews with ECEC staff in the same municipality where the main project was planned to be implemented. These interviews aimed to explore staffs’ perceptions regarding the needs related to a digital “healthy eating” resource. We used the Consolidated Criteria for Reporting Qualitative Research (COREQ) checklist [25] to ensure comprehensive reporting.

### Study sample

In Norway, there are approximately 5,343 ECEC centres, evenly divided between private and public ownership [26]. Each owner is responsible for staff competencies, training, and development. The municipal director oversees local ECEC centres and holds overall responsibility. Beneath the director are the managers of each individual ECEC, who are accountable for the competencies and development of their teachers and other staff [27]. The present study was conducted in a coastal, medium-sized Norwegian municipality (population 46, 000), with 400 births a year, and 33% of adult population having higher education of at least one year. The University of Agder and the municipality had an agreement to collaborate more closely on various areas, including research. This arrangement made it practical to test the digital resource in the municipality. All public and private ECEC centres (*n* = 59) were invited to participate in interviews during this phase of the *Nutrition Now* project.

### Recruitment and consent

Participants were recruited through the ECEC network of the municipality. In January 2020, the municipality’s director of ECEC organised a seminar where ECEC managers from all public and private ECEC centres were informed about the *Nutrition Now* project by the researchers. During this seminar, both the Director and the managers of the centres were present. The managers of each ECEC were invited to indicate their interest in receiving study-related information to the municipality’s Director of ECEC. Interested managers would subsequently receive information directly from the researchers, which they could then forward to their teachers. The researchers emphasised that both receiving information and participation were entirely voluntary.

Initially, eight ECEC teachers from seven private and one public ECEC were recruited to participate. Due to the uneven distribution of participation between private and public ECECs, the research team asked the Director of ECEC in the municipality to inform the managers about the study. The Director then verbally invited the managers of the municipal ECEC centres to participate, leading to the recruitment of four additional public ECECs in this round. Although the Director forwarded the contact details of interested managers to the research team, they did not know who ultimately participated. A total of 15 ECEC managers consented to be contacted and received an email containing information about the study and a consent form, which they could share with relevant ECEC teachers if desired. ECEC teachers interested in participating were then informed by their manager and received the consent form. Interested staff (teachers and managers) sent their completed forms directly to the researchers, ensuring privacy. At the end of each interview, participants were informed about the focus group invitation. An information letter and consent form for focus group participation, including consent for recording the sessions, were sent to the managers of the 15 ECECs.

### Data collection

#### Individual interviews

The individual interviews were conducted in two rounds: the first took place in May/June 2020, and the second in November 2020. This approach was chosen to ensure that perspectives from municipal ECEC centres were included, as initial participants were mostly from private centres. One of the authors (KV), who had no prior relationship with any of the participants, conducted all individual interviews at a convenient time during working hours for each ECEC staff member. Each interview lasted between 15 and 20 min, with additional time for a brief introduction explaining the study’s purpose and obtaining verbal consent. KV did not have previous experience with conducting interviews.

The guide for individual interviews covered ten topics, including: *(1) Level of knowledge about early-life nutrition among staff*,* (2) the current meal practice in the ECEC*,* (3) thoughts about integration of activities focusing on children’s relationship with food in ECEC*,* (4) thoughts about the use of a digital tool for accessing nutritional measure*,* (5) available cooking facilities in the ECEC centres*,* (6) involvement of children in cooking*,* (7) opportunities for informing and training staff*,* (8) ongoing pedagogical sessions and regular activities for children*,* (9) barriers related to implementing a digital learning resource*, and *(10) challenges in implementing food and meal measure for children*.

The interview guides used in this study have yielded multiple findings, and has been published in Helland et al. (2024) [23]. These findings have been crucial in planning the implementation strategies for the main project. This paper analyses six topics (1–5 and 10) relevant to its aims, while the remaining topics are covered in another article [23].

#### Focus groups

Two focus groups were conducted via Zoom in early December 2020. These sessions aimed to allow participants to discuss preliminary findings from the individual interviews with their peers, serving as a complementary step to the individual interviews. By reflecting on initial themes, we aimed to deepen our understanding of their perspectives and strengthen our initial findings through shared views during the group discussions.

Information letters were sent to the managers of 15 ECECs, who had agreed to receive project information, with the goal of recruiting 9 to 12 teachers or managers. This range was chosen to ensure a diverse sample for the focus groups and is considered optimal for in-depth discussions [28, 29]. Six managers accepted the invitation. One of the registered staff did not attend and did not provide a reason. The lower-than-expected response rate is likely due to the COVID-19 pandemic and the shift to Zoom, which may have impacted participation. To encourage active engagement and maintain discussion quality, participants were randomly divided into two small groups. KV, who had conducted all individual interviews and had no prior relationship with any of the participants, also led the focus groups. These sessions were held during working hours and lasted 53 and 39 min, respectively. KV introduced the study, obtained verbal consent, and guided the discussion.

Prior to the focus groups, interview data was preliminarily analysed and organised into the core components of the “healthy eating” resource: (i) the food environment in ECEC, (ii) educational activities involving food, (iii) mealtimes, and (iv) parental communication. A new theme, government guidelines and documents, was also added. The focus group guide covered: *(1) experiences with retrieving information from ECEC websites*,* (2) thoughts on the five core components of the upcoming digital “healthy eating” resource*,* (3) information needs of various user groups (managers*,* teachers*,* food managers*,* staff*,* children*,* and parents)*, and *(4) design and additional needs for the “healthy eating” resource.* The interview guide was published in Helland et al. (2024) [23]. Participants received a brief video summarising preliminary proposals to guide their reflections before the discussion.

### Data analysis

**Fig. 1:**
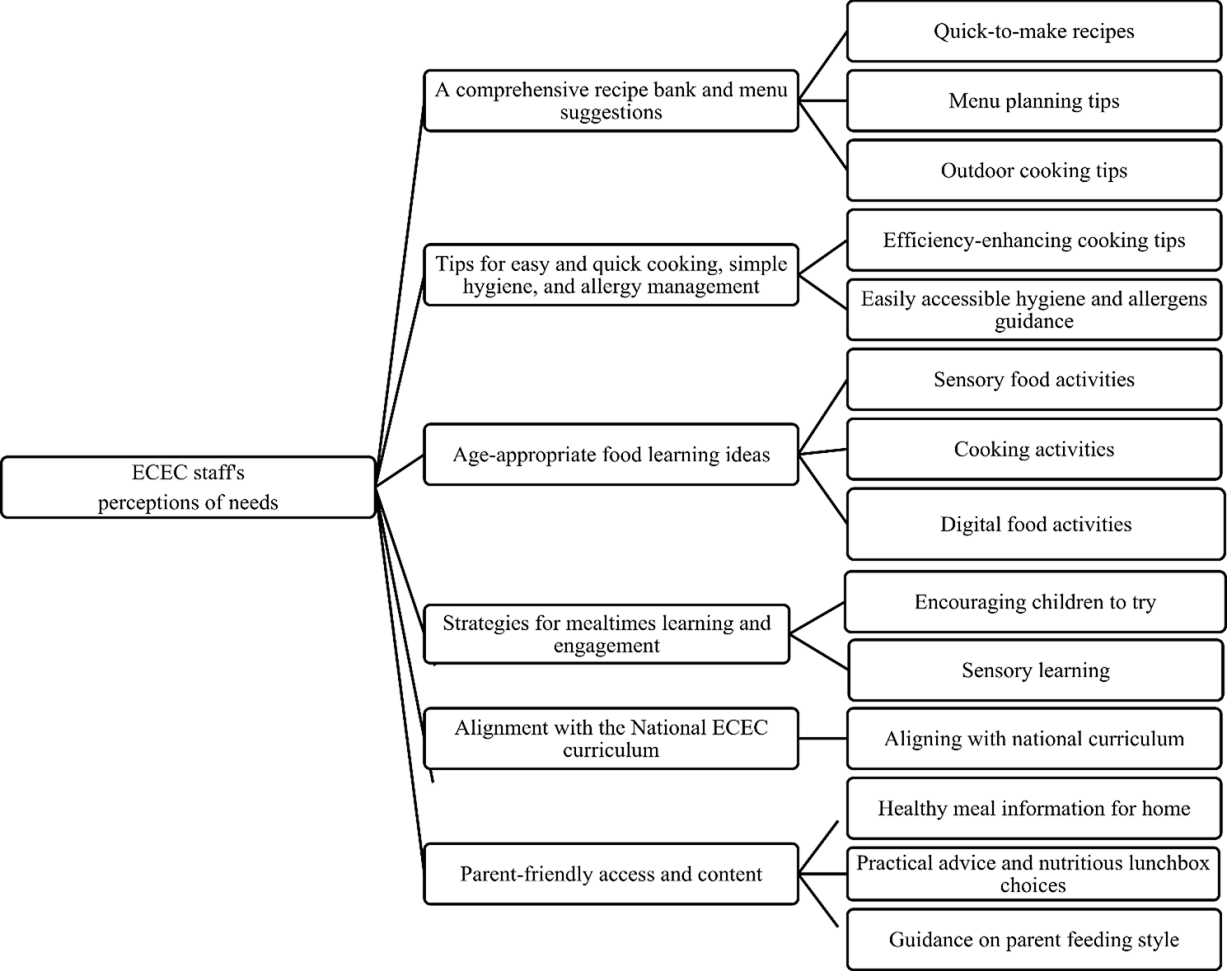
Main and sub-themes in ECEC staff’s perceptions of the features that guide and inform the development of a digital resource promoting “healthy eating” in ECEC settings

Interviews with ECEC staff were audio recorded and transcribed verbatim. The transcripts were analysed using thematic analysis, as developed by Braun and Clarke [30, 31]. Our primary focus was to identify key themes from the data, encompassing the most prominent and relevant aspects. We also recognised the importance of including themes that, while less frequently discussed by staff, were deemed significant for the enhancement of the “healthy eating” resource before its rollout to end-users. To ensure the study’s rigour, two authors (KV and SHH) independently coded all transcribed data using a coding framework based on the research question and the interview guide. The same analysis process was applied to the focus groups. The coding process was conducted in two stages. Initially, KV, a dietitian with a Ph.D., and a research assistant, a public health nutritionist with a master’s degree, coded the data in NVivo. Subsequently, SHH an ECEC teacher and chef with a Ph.D., re-coded the data independently in a separate process. SHH re-read all interviews to identify interesting excerpts, grouped the text into meaningful units, and applied appropriate codes. SHH then read through all the excerpts organised by code to gain a deeper understanding of each code, which were subsequently grouped into main themes and sub-themes. These themes were evaluated and revised to ensure distinctions between and within the main themes and sub-themes. SHH defined and named final themes, ensuring they aligned with the initial coding results from KV and the research assistant. Coding disagreements were discussed in a meeting. Codes were compiled into Fig. 1, with illustrative quotations from the transcripts in running text. The authors, including NCØ, a dietitian, and TB, a public health nutritionist both with Ph.D.s, used previous research, relevant theory, and their expertise to critically interpret the data for a deeper understanding of the findings. All authors discussed the interpretations, the need for reflexivity to enhance the study’s rigour. TB was the only man in the research team.

## Results

### Participants

A total of 12 out of 15 ECEC centres in the same Norwegian municipality consented to participate. This included seven private centres that provided some or all food on-site and five public centres where children typically brought food from home, except for one public centre that provided some food. Twelve ECEC teachers and one manager from 12 different centres participated in individual interviews, comprising twelve women and one man. Seven participants were from private ECECs and six from public ECECs. All participants had a minimum of three years of higher education. Additionally, two focus groups included five participants from four different ECECs, consisting of four women and one man. These participants represented three different public ECECs and one private ECEC. The staff who participated in the focus groups, holding the roles of manager or assistant manager, were not the same as those in the individual interviews. All had a minimum of three years of higher education. To distinguish individual ECECs when referencing quotes in the text, we have assigned each ECEC a distinct number within the range of 1 to 12, such as (I.12). For identifying separate focus groups, we’ve labelled them as FG.1 and FG.2.

### Main findings

The main findings of our study, based on thematic analysis, revealed six key themes (see Fig. 1) related to ECEC staff members’ perceptions of the necessary features for an upcoming digital resource to support staff in promoting healthy eating environments. To provide a more in-depth understanding, we present each theme along with relevant sub-themes and illustrative quotes in the following text.

#### Theme 1: a comprehensive recipe bank and menu suggestions

This theme highlights ECEC staff members’ preferences for a diverse, user-friendly recipe bank and menu resource to support the implementation of the upcoming “healthy eating” resource, catering to various age groups and nutritional guidelines.

##### Quick-to-make recipes

Staff members expressed a desire for simple, efficient recipes that save time. They wanted recipes that are quick to prepare, suitable for larger groups, and nutritionally adequate: “So, something that’s efficient to make, …with fewer cooking steps, things that you can prepare in large quantities and that are quick while still providing necessary nutrition” (FG.1).

##### Menu planning tips

One staff member emphasised the need for menus that include recipes and shopping lists to ensure a diverse and well-planned diet.

*It would be great*,* actually very convenient*,* to have a menu plan. That way*,* we wouldn’t need to think about what to serve*,* write shopping lists*,* and all the fuss about what fish dinner to make. A more organised*,* well-prepared shopping list would be nice* (I.3).

Another staff member noted the current approach, where the chef relies on available ingredients and lacked systematic planning: “She just goes with what she has available… I’ve mentioned that this is your job, and I’m not taking responsibility for it” (I.4). During focus groups, one staff member shared a positive experience from a previous workplace: “… we had a person in charge of food, and she created three-month menus” (FG.2). Another staff member wanted separate menus for the youngest children to ensure age-appropriate meals.

##### Outdoor cooking tips

This was of interest due to the frequent forest excursions in Norwegian ECECs. One staff member (I.3) suggested adding outdoor cooking recipes to the resource. Two staff members in a focus group explained: “The outdoor food aspect, I think, is also quite important. What’s suitable to make outdoors.” The other staff member stressed the need for outdoor planning tips: “…like when you’re going on a forest trip and planning to have vegetable soup— remember to chop the vegetables the day before” (FG.2).

#### Theme 2: Tips for easy and quick cooking, simple hygiene and allergy management

Staff members emphasised the need for streamlining cooking processes and improving time efficiency. Simultaneously, they highlighted the significance of user-friendly safety instructions for managing allergens and intolerances to ensure children’s well-being.

##### Efficiency-enhancing cooking tips

Staff members stressed the need for quick cooking tips, such as using pre-cut or frozen vegetables. One staff member shared an experience.

*I had employees who were peeling potatoes almost to the point of their fingers bleeding. I thought*,* ‘Okay*,* can’t we find a simpler way?’ I mean*,* we need to find those simple conveniences [like pre-peeled potatoes] that don’t compromise quality. I believe that’s one of the key features when we enter [the digital resource]*,* and it should be a tool where you feel you find what you need when you’re preparing food in the ECEC* (FG.1).

Another staff member stressed the need for planning tips to reduce cooking stress and enhance the resource’s utility: “…make it easy… with things you can prepare before leaving work…. If you can find it on that site [in the digital resource], I believe it’s going to be used a lot…” (FG.1). Additionally, a staff member proposed labelling recipes with difficulty levels and time estimates: “…[If] I’m told to make a fish dish for Thursday, I can …enter the number of children and get an estimated preparation time” (FG.1).

##### Easily accessible hygiene and allergens guidance

Some staff members wanted clear and concise guidelines on food handling, hygiene, and allergens in the digital resource. One staff member (I.6) underscored the need for insights into clean and unclean zones and National Food Safety Authority regulations: “…what’s acceptable and what’s not, so to speak, in relation to clean and unclean zones”. Another staff member emphasised the challenge of understanding food storage and cooking regulations: “…understanding what the Norwegian Food Safety Authority requires for food preparation is a job in itself. Food storage, in general, everything around food handling” (I.10).

Both focus groups discussed food allergies and intolerances, with staff requesting practical tips on managing allergens: “Tips regarding allergies and such would be really great… Something easy to look up” (FG.1). They also mentioned the need for guidance on suitable alternatives for lactose intolerance (FG.2).

#### Theme 3: age-appropriate food learning ideas

This theme underscores staff members’ enthusiasm for incorporating age-appropriate food-related educational activities. One staff member remarked, “Yes, absolutely. There’s an interest in trying out activities related to food. We’re always open to new things” (I.8). Another staff member said: “Definitely, if we can get some good ideas and exchange information” (I.9). Another added: “Yes, but a website where one can gather tips and ideas, resources, and what to do with children, that would be fantastic” (I.10). One staff member highlighted the need for convenient access to activities tailored for children aged one to three, explaining: “It must be designed so that, [for example], if you have a toddler section, you can easily figure out and press some buttons to make it readily accessible for what you actually desire and achieve” (FG.1).

##### Sensory food activities

One staff member expressed interest in sensory food activities but was uncertain about their execution. When asked if she needed specific assistance from the upcoming digital resource for implementing such activities, she replied with a sigh: “No, I don’t know what it should be” (I.09). During a focus group, another staff member suggested educational activities involving vegetables, such as sensory exploration and harvesting, with discussions about taste, smell, and hands-on experiences like harvesting corn or carrots (FG.1).

##### Cooking activities

Staff emphasised the importance of involving children in cooking to increase their willingness to try new foods. One staff member stated, “If the children are involved, it’s much easier for them to eat it afterwards. If everyone sits and tries and tastes, it can become interesting for the others” (I.8). Another staff member suggested the digital resource should include ideas for involving children in cooking activities, such as “what the children can participate in” and “what you can prepare the day before” (FG.2). This guidance can help employees plan and organise activities involving children.

A staff member in FG.2 proposed cooking videos to show how to cook with children to enhance the resource’s use. In individual interviews, two staff members expressed interest in ideas for involving children under three years in cooking. One staff member (I.7) recognised the difficulty of engaging younger children, while another expressed curiosity about including them: “Typically, it’s often those, maybe from three years and older, who are most involved in cooking. But it would be fun to find a way to include the younger ones as well” (I.3).

##### Digital food activities

Staff expressed interest in integrating digital food-related activities into the resource. One idea was an interactive e-book with clickable vegetable icons: “…something that the children can see and interact with, perhaps with some vegetable icons” (I.3). Another suggestion was to offer digital content that actively engages children in food-related learning, making education seamless without extensive effort: “… It’s a natural way of learning as well, without having to do much more than just showing and talking about what we’re doing” (I.2).

#### Theme 4: strategies for mealtime learning and engagement

Although only a couple of staff members raised topics related to mealtimes, their discussions highlighted the potential of the upcoming resource to provide information on using mealtime as an opportunity for children to develop a preference for various foods.

##### Encouraging children to try

One staff member emphasised the importance of providing staff with information on engaging ways to encourage children to try new and unfamiliar foods during mealtimes.

*It is important that… everyone has access to the same information. …introducing new food to children can be done in an exciting way*,* to make them want to taste it and find it enjoyable. Because if they just get a vegetable thrown on their plate*,* it is not always fun to taste it* (I.8).

##### Sensory learning

One staff member suggested incorporating sensory learning as an educational tool during mealtimes: “Mealtimes can indeed be an educational situation, by discussing the food, how it tastes, what it looks like, [and its] colours… that my [food] is different from yours” (I.12).

#### Theme 5: alignment with the national ECEC curriculum

Staff highlighted the value of aligning the resource with the national ECEC curriculum to streamline documentation. One staff member noted, “Some find it very satisfying to have a setup where everything is kind of laid out… what you need, what to do, how it’s connected to the curriculum” (I.1). This alignment helps staff document their work and ensure curriculum compliance, which is especially useful for pedagogical leaders. They actively use the curriculum for documentation purposes (FG.2).

Two staff members proposed that showing how ECEC practices align with the national curriculum might interest parents and support staff. Making this information available might help parents better understand the ECEC’s teachings and care and offer staff a sense of accountability:

*…. If parents gain access*,* they can go in and see—well*,* maybe they’re interested in some aspects of what’s in the curriculum. I think that’s nice if there’s also something you can click on if you’re interested* [for more details] (FG.2).

#### Theme 6: parent-friendly access and content

Staff believed the resource could be a powerful tool for engaging parents in their children’s health and nutrition education. As noted by one staff member, “It will be good and useful for the parent group too. Do they have access to the same resource?” (I.11).

##### Healthy meal information for home

ECEC staff stressed the need for collaboration between staff and parents to promote healthy eating. As one staff member noted, “if parents are on board, much of the job is done” (I.2). Another staff member underlined the significance of a digital resource for parents: “I think the most important thing is outreach to parents” (I.11). The resource was considered an eye-opener for parents, with one said: “Maybe parents will notice at home that their children are more interested in vegetables or asking for new foods” (I.3). Staff recognised the potential of a digital resource to enhance parents’ understanding of healthy food. They also suggested providing ECEC’s healthy recipes online for parents, noting challenges related to cultural diversity and some families’ limited understanding of healthy food (I.7).

##### Practical advice and nutritious lunchbox choices

Staff members recommended providing practical advice for parents on packing nutritious and diverse meals for ECEC. This could include tips for making healthy lunchboxes: “Some practical tips for including [proper food] in the ECEC lunch, like specific things to consider. And, as part of parent communication, we could share that here [the digital resource]” (FG.2). They found it helpful to refer to a website to refer to during parent-teacher meetings, especially for children with limited diets: “Some [children] have a very one-sided diet. So, we sometimes address this in parent-teacher meetings, and it’s really nice to have a website to refer to” (FG.2).

##### Guidance on parent feeding style

In a focus group, staff emphasised the need for guidance on fostering positive mealtimes at home through the digital resource. They suggested providing advice on establishing favorable mealtime habits, such as family dining, serving the same food to all, and handling selective eating. Staff observed that some parents struggle with mealtime boundaries, which can lead to children not eating at the table: “…We’ve had to guide parents several times on the fact that it’s not just children eating alone in front of a screen. … There are some parents who find it challenging, this matter of food and setting boundaries” (FG.2). The resource could support parents in creating a positive mealtime environment, especially for picky eaters. Many parents seek advice on handling picky eaters: “It’s a somewhat typical question parents ask” (FG.2). This support can boost confidence in promoting healthy eating habits.

## Discussion

This study explores the perceptions of staff working in Norwegian ECECs regarding the needs related to the content of an upcoming digital “healthy eating” resource. Overall, staff seem to require support and resources to effectively promote healthy eating in ECEC settings, a need that aligns with the broader aims of Wallace et al. (2017), who also sought to understand staff members’ needs in this context [32]. We will relate the results illustrated in Fig. 1 to the following four core components: *ECEC environment*, *teacher/staff components*, *child components*, and *parental components* in line with Matwiejczyk et al. (2018). These insights can help enhance the user-friendliness of such a digital resource for ECEC staff, thereby facilitating broader implementation in the future.

### The food environment in ECEC

plays a crucial role in shaping healthy eating habits among children. Effective ECEC programmes aimed at promoting healthy eating habits involve making environmental changes such as menu modifications, policy adjustments, and changes to food provision [6]. Since staff members play a key role in implementing food-related environmental factors, the upcoming digital “healthy eating” resource should include a “food preparation” component tailored to meet their needs. In our current study, staff underscored their preferences for several specific features in this core component, namely: (1) the inclusion of age-appropriate recipes and (2) menu plans aligned with national recommendations, (3) straightforward instructions for both indoor and outdoor cooking, (4) guidance for replacing allergens, (5) tips for efficient and safe cooking practices, and (6) the incorporation of official food safety requirements into the digital resource.

Grady et al. found that a web-based menu-planning intervention significantly increased servings of fruit, vegetables, dairy, and meat while reducing discretionary foods at 3 months post intervention, with some improvements sustained at 12 months [33]. Similarly, Devine et al. (2019) demonstrated that online programmes can lead to systematic changes in the food environment by improving knowledge and practices among ECEC staff [34]. These findings suggest that incorporating these targeted features into the digital resource might enhance its effectiveness.

In our previous version of the digital “healthy eating” resource, which was implemented in Norwegian ECEC over three months, we emphasised age-appropriate recipes and menu plans (features 1 and 2 above) [21]. This intervention positively impacted children’s vegetable intake [22], and closely matches the preferences of the teachers in the current study for the food environment component. Nevertheless, we recognised the need for additional information and further simplification of food preparation procedures to better support the teachers in implementing healthy eating practices in ECEC, especially in settings without a chef on staff. Incorporating instructional videos and text on simple food preparation methods appears to be a valuable addition to the “healthy eating” resource.

### The child component

A multi-component approach that includes both educational components for children (such as age-appropriate materials and activities) and ECEC’s food environment is shown to be effective in promoting healthy eating habits among children [6]. The findings of this study indicated that teachers are interested in incorporating age-appropriate hands-on activities related to food preparation and food and sensory learning into their educational practices with children. The results emphasise the need for activities specifically adapted to children under the age of three, which is in line with previous research on how young children learn about food [35]. However, many expert-led interventions do not include these types of activities, which may limit their effectiveness [36].

Our previous version of the digital “healthy eating” resource, a multi-component intervention study conducted in Norwegian ECEC, emphasised age-appropriate playful sensory lessons [21], closely aligning with the perceptions and needs of the teachers in the current study regarding pedagogical components. Two of our Norwegian studies suggest that sensory learning sessions may have a positive impact on younger children’s attitudes and behaviours towards healthy eating. While more research is needed to confirm these findings, incorporating sensory learning activities into digital “healthy eating” resources may be a promising approach to promoting healthy eating habits among young children [18, 19]. Participants suggested using interactive e-books with clickable vegetable icons to engage children in food-related learning; while such activities can effectively engage children, it is important to ensure they align with guidelines for limiting screen time in this age group. By providing hands-on, sensory learning experiences related to healthy eating, we might help young children develop positive attitudes and behaviours towards new and novel food [19], which has the potential to lead to healthier eating habits in the long run [3, 37].

### The teacher/staff component

ECEC plays a crucial role in shaping healthy dietary habits in children by providing a supportive social and physical environment. Typically, dietary programmes target the behaviours and practices of ECEC teachers and staff and involve education or training sessions [6]. In our current study, staff primarily focused on the *food environment in ECEC settings*, and placing less emphasis on staff practices during mealtime situations. However, two of the staff noted that increasing children’s willingness to try new foods and integrating sensory learning into mealtimes are important and should therefore be topics included in the digital resource. These findings should be interpreted with caution, as the way the interviews were conducted may have influenced them.

The lack of emphasis on mealtime practices may stem from gaps in the Norwegian teacher education curriculum, which does not prioritise development of healthy eating habits [38]. A summary of Scandinavian studies show that teachers struggle with defining educational goals for mealtimes, highlighting the need for clear objectives to promote health and collaboration among staff and different groups of staff [39].

Our previous version of the digital “healthy eating” resource, included a couple of videos on promoting healthy eating [21], aligned with Haines et al. (2019) recommendations for positive parental feeding practices. These practices include avoiding food restriction, allowing children autonomy in food choices, promoting portion control, eating together, modelling healthy eating, and creating enjoyable mealtime environments [40]. Due to the teachers’ limited focus on staff mealtime behaviour, and the key role of ECEC staff in supporting food sharing and family-style meals [41], we aimed to strength this core component in three ways: by adding a section on healthy mealtime practices, creating printable and posters to display in the ECEC settings, and incorporating a reflection template for internal staff meetings to encourage professional development. Insights from the “Supporting Nutrition in Australian Childcare” (SNAC) project and their findings related to the development and implementation of a digital nutrition education programme highlight the challenges in achieving lasting changes among ECEC staff. Wallace’s (2016) argues that broader policy changes are needed to prioritise food and mealtimes in ECEC settings. They also emphasise that achieving sustained improvements requires more than just digital programmes [42]. ECEC staff in the current study suggested linking the resource with the national curriculum for ECEC [20], as well as recommendations from health authorities regarding food and mealtimes in ECEC settings [14]. We have now integrated detailed descriptions into the resource, demonstrating how staff can effectively address diverse learning objectives by implementing a digital “healthy eating” resource. These descriptions can serve as valuable documentation for ECEC mealtime efforts. Further exploration is needed to determine the best methods for supporting implementation and bridging the gap between the two disciplines: early childhood education and health promotion [43, 44].

### The parental component

Including a parental component is recommended in “healthy eating” programmes designed for ECEC [1]. Even minimal parent involvement, such as receiving written material, has been linked to more positive dietary outcomes for children [6]. In our present study, staff expressed the desire for parents to have access to the upcoming digital “healthy eating” resource. Staff believed that building partnerships with parents through digital resources would support their work. This aligns with a U.S. study emphasising the importance of engaging parents in promoting children’s nutrition. Strategies include parental nutrition education in improving children’s health outcomes and fostering respectful partnerships between staff and parents [45]. Our plan reflects this approach, with the upcoming digital resource consisting of two equal parts: one for parents and one for ECEC staff [24]. In addition, we incorporated a dedicated section that provided staff with valuable tips for facilitating conversations with parents, in line with Dev et al. (2017) [45]. This includes documenting the ECEC’s food environment, feeding styles and practices, as well as offering tips on how to collaborate with parents regarding individual children in ECEC [44]. Furthermore, we encouraged the staff to motivate parents to use the parent section. By expanding the programme to include both the home and ECEC settings, healthy food preferences and dietary-related behaviours can be influenced more effectively than by ECEC alone [1, 6]. Given the limited focus on food habits and preferences in the Norwegian teacher education programme [37], it is essential to address the need for support and training to improve staff members’ self-efficacy in engaging parents and effectively conveying key messages about healthy eating. Research indicates that a lack of skills, knowledge, and confidence in communicating with parents regarding healthy eating may mean that the potential for implementing healthy eating strategies is not being fully realised [46].

### Strengths and limitations

Our study involved multiple teachers, including five who also served as managers of ECEC centres where the resource was intended to be implemented. This diversity provided a range of perspectives, including those from ECECs where lunch boxes are brought from home and where staff have limited experience with cooking. By using both interviews and focus groups, we were able to collect more in-depth data, which enabled this information to be presented more succinctly. The focus groups facilitated the exchange and comparison of experiences and allowed participants to share their perspectives in a small group setting, ensuring that each voice was heard. All interviews were conducted by one of the authors not involved with the existing digital resource, which reduced the potential for bias. To enhance the rigour of the analysis, three researchers with different backgrounds independently coded the interviews initially.

Conducted in a Norwegian context, the findings may not be directly applicable to other settings due to differences in public arrangements and national guidelines. Future research should explore diverse countries and professional groups, such as chefs or individuals with lower levels of education, to gain broader insight into the needs for core components of a digital healthy eating resource. Recruitment was uneven, and the second-round recruitment may have included participants who were less motivated. Additionally, staff were not shown the existing digital resource, which may have limited their feedback. The limited number of focus group participants due to the COVID-19 pandemic may have reduced group dynamics and the richness of the data. Additionally, the absence of previously interviewed participants may have hindered deeper exploration of the themes, potentially affecting the credibility and transferability of the findings.

## Conclusion

ECEC staff in this study highlighted a range of topics relevant to include in a new digital “healthy eating” resource. These topics appeared pertinent to their daily work with food and mealtimes and included easy-to-prepare menus, age-appropriate food-related activities for children, alignment with the national ECEC curricula and, empowering parents with knowledge of food and mealtime practices. Notably, the staff reflections primarily centred around cooking, with few addressing broader mealtime settings. This limited consideration of mealtimes as a significant opportunity for children’s learning and the development of healthy habits likely reflects the absence of a strong connection between pedagogy and health in this field. Thus, it underscores the importance of including this topic in the digital “healthy eating” resource for ECEC settings. The insights gained from this study were used to improve the relevance and user-friendliness of a digital “healthy eating” resource, which plays a crucial role in the “scale up” of the *Nutrition Now project*.

## Data Availability

The data will be available to other researchers for non-commercial purposes upon request to the corresponding author.

## Abbreviations

ECEC: Early childhood and care
